# Comparison of generic and lung cancer-specific quality of life instruments for predictive ability of survival in patients with advanced lung cancer

**DOI:** 10.1186/s40064-016-3492-7

**Published:** 2016-10-21

**Authors:** Sultan Eser, Tuncay Göksel, Ahmet Emin Erbaycu, Hakan Baydur, Burcu Başarık, Ayşen Öz Yanık, Kader Kıyar Gürsul, Pınar Çelik, Ebru Çakır Ediz, Osman Hatipoğlu, Bedriye Atay Yayla, Sevin Başer, Erhan Eser

**Affiliations:** 1 Institute of Public Health, Hacettepe University, Ankara, Turkey; 2 Department of Chest Diseases, Faculty of Medicine, Ege University, İzmir, Turkey; 3 Dr. Suat Seren Chest Diseases and Thoracic Surgery Education and Research Hospital, İzmir, Turkey; 4 Department of Social Work, Faculty of Health Sciences, Celal Bayar University, Manisa, Turkey; 5 Department of Chest Diseases, Faculty of Medicine, Gazi University, Ankara, Turkey; 6 Department of Chest Diseases, Nevsehir State Hospital, Nevsehir, Turkey; 7 Department of Chest Diseases, School of Medicine, Ege University, İzmir, Turkey; 8 Department of Chest Diseases, School of Medicine, Trakya University, Edirne, Turkey; 9 Department of Chest Diseases, School of Medicine, Pamukkale University, Denizli, Turkey; 10 Department of Public Health (Halk Sağlığı AD), School of Medicine (Tıp Fak), Celal Bayar University, 45040 Manisa, Turkey

**Keywords:** Lung cancer, HRQOL, Prognostic factors, Survival

## Abstract

**Background:**

Our purpose is to examine the relationship of Health related quality of life measured by EORTC QLQc30, QLQ-LC13; FACT-L, LCSS, Eq5D) with survival in advanced lung cancer patients. A total of 299 Lung Cancer (LC) patients were, included in this national multicenter Project entitled of “the LC Quality of Life Project (AKAYAK). Baseline scores were analyzed by using Cox’s proportional hazard regression to identify factors that influenced survival. Univariate and multivariate models were run for each of the scales included in the study.

**Results:**

Mean and median survival were 12.5 and 8.0 months respectively. Clinical stage (as TNM), comorbidity; symptom scales of fatigue, insomnia, appetit loss and constipation were associated with survival after adjustment for age and sex. Global, physical and role functioning scales of QLQc30; physical and functional scales of LCS and TOI of the FACT-L was also associated with survival. Mobility and Usual activities dimensions of the Eq5D; Physical functioning and the constipation symptom scale of the QLQ-c30; and LCS and TOI scores of the FACT-L remained statistically significant after adjustment. LC13 and LCSS scales were not predictors of survival.

**Conclusions:**

HRQOL serves as an additional predictive factor for survival that supplements traditional clinical factors. Besides the strong predictive ability of ECOG on survival, FACT-L and the Eq5D are the most promising HRQOL instruments for this purpose.

## Background

Health-related quality of life (HRQOL) (Coates et al. 1997) domains have been valued mainly when survival gain in clinical trials remains unanswered (Dancey et al. 1997), but its use is very restrictive for clinical decision making (Vigano et al. 2004).

Lung cancer (LC) is one of the common cancers in men worldwide, and survival, treatment modalities, and HRQOL of LC patients are issues that need to be addressed in a clinical context. It is both an important and difficult task for clinicians to predict prognosis in cancer patients and especially for patients having advanced lung cancer. Until recent decades, health professionals used objective performance indicators in a dominant role to predict the prognosis of lung cancer. Several studies have shown the ability of HRQOL instruments to predict survival based on different cancer sites (Coates et al. 1997; Dancey et al. 1997; Vigano et al. 2004; Gotay et al. 2008; Quinten et al. 2009, 2014; Grande et al. 2009; Montazeri et al. 2001; Li et al. 2012; Langendijk et al. 2000; Efficace et al. 2006; Maione et al. 2005; Herndon et al. 1999; Polanski et al. 2016; Ganz et al. 1991; Eton et al. 2003; Bernhard et al. 1996; Dharma-Wardene et al. 2004; Reck et al. 2012; Hwang et al. 2004; Sloan et al. 2012; Qi et al. 2009; Kaasa et al. 1989; Naughton et al. 2002; Nowak et al. 2004; Braun et al. 2011; Nishiyama et al. 2006; Movsas et al. 2009). These studies, either reviews (Gotay et al. 2008; Mannion et al. 2014; Guyatt et al. 1993) or longitudinal design studies, found that baseline HRQOL was a prognostic indicator of survival. In their recent review, Mannion et al. (2014) stated that the European Organisation for Research and Treatment of Cancer-Quality of Life Questionnaire (QLQ-C30) was the most widely used questionnaire; other commonly used scales include the Functional Assessment of Cancer Therapy-Lung (FACT-L) and the Lung Cancer Symptom Scale (LCSS). Although there is a consensus on the assessment of HRQOL in predicting survival in LC patients, it is still unclear which of these instruments better predicts survival. Until recently, there have been very few studies that aimed to compare different HRQOL instruments with regard to their ability to predict survival in LC patients. Only one study (Grande et al. 2009) used generic QOL measurement tools to predict survival of patients having lung cancer. A disease-specific measure may provide more detailed outcome information and so may be more relevant to patients and clinicians (Guyatt et al. 1993), although it was stated that the overall impact of functioning and well-being may be missed by using only a disease-specific measure (Coons and Shaw 2005).

The objective of this study was to examine the prognostic value of baseline HRQOL for survival in any type of LC using well-known self-assessment tools of HRQOL in lung cancer. To our knowledge, this multi-centre study conducted the first analyses of QOL as a prognostic factor for survival among patients having all types of lung cancer, by using of a battery of different generic, cancer- and lung cancer-specific QOL instruments plus performance status.

## Methods

### Study design

This study was performed within the framework of a national multi-centre project entitled “The Lung Cancer Quality of Life Project” (AKAYAK-1). We contacted 299 LC patients undergoing active chemotherapy, surgery, or post-therapy follow-up from April 2010 to February 2012 in an inpatient setting or at the outpatient clinics of five comprehensive cancer centres in western Turkey

HRQOL and performance scales were completed at baseline for all patients regardless of the treatment type (chemotherapy, radiotherapy or a combination).

During these visits, HRQOL instruments were applied to the patients via interviewer assistance and the Karnofsky performance status (KPS) and Eastern Cooperative Oncology Group (ECOG) performance status (Oken et al. 1982) were also assessed for all patients by nurses/physicians.

#### Compliance with ethical standards

The AKAYAK project has obtained an ethical approval by the Research Ethics Committee of Ege University, Izmir, Turkey. The authors of this manuscript have no affiliations with or involvement in any organization or entity with any financial interest, or non-financial in the subject matter or materials discussed in this manuscript. No funding was received for this study.

### Patients

The inclusion criteria for the patients (*n* = 299) in this trial were as follows:

- Patients who were diagnosed with Primary LC as stage IIIB or IV (including all histological types).
- Age between 18 and 76 years.
- Previously untreated and planning to undergo chemotherapy, radiotherapy, or chemo-radiotherapy.
- Ability to read and complete questionnaires,
- Agreed to participate in the study and volunteer to attend the control visits.
- Written informed consent form was provided.

### HRQOL scales and variables examined

#### HRQOL scales

##### “The European Organisation for Research and Treatment of Cancer Quality of Life Questionnaire (EORTC QLQ-C30)”

“EORTC QLQ-C30 consists of 30 items assessing global HRQOL. These items are grouped into five functional scales, including physical functioning, role functioning, emotional functioning, cognitive functioning and social functioning; into three symptom scales including fatigue, nausea and vomiting, and pain; and six single-item scales including dyspnoea, insomnia, appetite loss, constipation, diarrhoea, and financial difficulties” (Aaronson et al. 1993). A validated (Guzelant et al. 2004) Turkish version of the QLQ-C30 was used in this study.

##### “EORTC Lung Cancer Scale (QLQ-LC 13)”

“The QLQ Lung Cancer module (QLQ-LC13) consists of 13 questions assessing lung cancer-associated symptoms (cough, haemoptysis, dyspnoea, and site-specific pain), treatment-related side effects (sore mouth, dysphagia, peripheral neuropathy, and alopecia), and pain medication” (Bergman et al. 1994). QLQ LC13 was validated into Turkish by Ataman et al. (2008).

##### Functional assessment of cancer therapy-lung cancer (FACT-L)”

Turkish version of the FACT-L was developed by Basarik (2011). FACT-L is a combination of a generic cancer scale, the Functional Assessment of Cancer Therapy-Generic (FACT-G) and a lung cancer subscale (LCS), and consists of 37 questions (Cella et al. 1993, 1995). Generic subscales and the item compositions of FACT-G are as follows: Physical Well-Being (seven items), Social/Family Well-Being (seven items), Emotional Well-Being (six items), and Functional Well-Being (seven items). The 10-item LCS assesses LC symptoms. The Trial Outcome Index (TOI) is a 21-item single-score scale that sums the PWB, EWB, and LCS subscales of the FACT-L, proposed to assess the physical components of HRQOL. Total possible scores range between 0 and 84, with higher scores indicating a better QOL” (Cella et al. 1995).

##### Lung Cancer Symptom Scale (LCSS)

The LCSS evaluates six major symptoms associated with lung malignancies and their effects on overall symptomatic distress, functional activities, and global QOL. Physical and functional dimensions are assessed by five items, and an average of the aggregate score of all nine items is defined as the total score (Hollen et al. 1995). Korkmaz and Fadiloglu (2007), developed and validated Turkish version of the LCSS in 2007.

##### EuroQoL (EQ5-D)

EQ 5-D was developed as a five dimension preference-based measure of HRQOL (EuroQol_Group 1990). In this study, we used the validated Turkish version (Eser et al. 2007) of the index.

#### Prognostic clinical variables

Tumour characteristics, such as histology, tumour type, stage, duration of illness, type of treatment, and patient characteristics, including age, gender, education, and co-morbidities at the time of diagnosis, were obtained. All histological types were included in our study. The 7th edition of lung cancer TNM classification and staging system was used in this study (http://www.uicc.org/resources/tnm/publications-resources).

### Statistical analyses

Baseline HRQOL and performance assessments were used for predicting survival in this study. Initially, the scales of the QLQ C-30, LC-13, FACT-L, and LCSS were categorised according to tertiles, whereas the single-item symptom scales of QLQ C-30 and EQ5-D dimensions were dichotomised. In univariate Cox analysis, baseline HRQOL scores were used as independent variables to assess the crude risk of survival separately for each scale. A multivariate Cox’s regression analysis was performed by adjusting baseline scores on global QOL for known prognostic factors such as age, gender, clinical stage, and co-morbidities.

In addition to this multivariate analysis that only adjusted for the effects of these known prognostic factors, we examined six multivariate models indicating the relative hazards for survival for demographic and clinical variables and HRQOL scales. The model A showed the relative hazard for survival for only the demographic and clinical variables that were found significant in the univariate analyses. In the model B, the functioning scales of the QLQ C30 that were found significant in the univariate analyses which were examined simultaneously by adjusting for the demographic and clinical variables entered into the model A. “The model C” differed from the second by entering the symptom scales of the QLQ C30, instead of the functioning scales of the QLQ C30. In the model D, we combined models B and C. In the model E, the relative hazards of survival were estimated simultaneously for significant dimensions of the Eq5-D by controlling demographic and clinical variables. Finally, the FACT-L scales that were found to be significant in the univariate analyses were examined in the final model (Model F).

Survival curves were estimated using the Kaplan–Meier method. The log-rank test was used to determine the statistical significance of the differences between curves. The level of significance was set at 0.05. Statistical analyses were performed using the SPSS software (version 15.0 for PC).

## Results

All patients (n = 299) had TNM system stage III (38.5 %) or IV (61.5 %) disease and an ECOG performance status of 0 or 1 (70.9 %) at baseline (Table 1). Univariate hazard ratios (HR) indicated shorter survivals for female gender (HR 1.74, 95 % CI 1.06–2.85); higher stage (stage IV) (HR 2.13, 95 % CI 1.64–2.77); distant metastasis (HR 1.68, 95 % CI 1.31–2.15); presence of co-morbidities (HR 1.49, 95 % CI 1.16–1.91); non-surgery treatment experiences (chemotherapy: HR 1.35, 95 % CI 1.02–1.80; radiotherapy: HR 2.11, 95 % CI 1.44–3.08; adjuvant therapy: HR 2.89, 95 % CI 1.60–5.22); and patients who did not undergo pneumonectomy/lobectomy (HR 3.55, 95 % CI 1.13–11.18). Age, the level of education, and tumour type had no significant impact on survival.

**Table 1:** The relation to demographic and illness related characteristics of Lung Cancer patients to survival time

Variables	N (%)	HR (95 % CI)
Gender		
Male	23 (7.7)	1.74 (1.06–2.85)*
Female	276 (92.3)	1.00
Age (years)		
<50	36 (12.0)	1.00
50 ≤ Age < 60	114 (38.1)	0.94 (0.63–1.40)
60 ≤ Age < 70	101 (33.8)	1.13 (0.76–1.69)
≥70	48 (16.1)	1.53 (0.97–2.41)
Education		
İlliterate–primary	223 (75.1)	1.00
Secondary and over	74 (24.9)	0.83 (0.63–1.09)
Type of cancer		
Adenocarcinoma	69 (23.2)	1.00
Squamous cell	110 (36.9)	0.95 (0.69–1.31)
Small cell	61 (20.5)	0.84 (0.58–1.21)
Other	58 (19.5)	1.21 (0.84–1.76)
Clinical stage		
Stage 3B	115 (38.5)	1.00
Stage 4	184 (61.5)	2.13 (1.64–2.77)***
Distant metastasis		
Yes	156 (52.2)	1.68 (1.31–2.15)***
No	139 (46.5)	1.00
Comorbidity		
Yes	103 (34.4)	1.49 (1.16–1.91)**
No	196 (65.6)	1.00
Pneumectomy/lobectomy		
Yes	10 (3.3)	1.00
No	289 (96.7)	3.55 (1.13–11.18)*
Chemotherapy		
Yes	230 (76.9)	1.35 (1.02–1.80)*
No	69 (23.1)	1.00
Adjuvan therapy		
Yes	19 (6.4)	2.89 (1.60–5.22)***
No	280 (93.6)	1.00
Radiotherapy		
Yes	35(11.7)	2.11 (1.44–3.08)
No	264 (88.3)	1.00

* P < 0.05; ** P < 0.01; *** P < 0.001

Tables 2, 3 and 4 show the results of univariate and multivariate analyses: In Table 2, global QOL, physical functioning, role functioning scales, and symptom scales for fatigue, insomnia, appetite loss, and constipation from QLQ-C30 were associated with survival after adjustment. None of the scales of the QLQ-LC13 or the index or symptom scores of LCSS showed a significant effect on survival even in univariate analyses (Table 3).

**Table 2:** Significance of QLQ-C30 scores for overall survival of the lung cancer patients (Univariate and mulitvariate Cox’s regression results)

Variables	Mean (SD)	N	Crude HR (95 % CI)	Adjusted HR^a^ (95 % CI)
Global QoL (≥66.67 as reference)	57.6	134	1.00	1.00
50.00 > GL ≤ 66.66	(24.3)	84	1.09 (0.79–1.52)	1.02 (0.73–1.43)
≤50,00		81	1.57 (1.17–2.12)***	1.39 (1.03–1.88)*
Physical functioning (≥86.67 as reference)	70.4	115	1.00	1.00
66.66 > PF ≤ 86.66	(27.7)	102	1.27 (0.93–1.75)	1.11 (0.80–1.54)
≤66.65		82	2.09 (1.53–2.85)***	1.65 (1.19–2.29)**
Role functioning (≥100.0 as reference)	70.7	151	1.00	1.00
66.66 > RF ≤ 99.99	(32.4)	22	1.39 (0.87–2.21)	1.32 (0.81–2.16)
≤66.65		126	1.56 (1.21–2.02)**	1.36 (1.04–1.78)*
Emotional functioning (≥91.66 as reference)	77.8	103	1.00	1.00
66.66 > EF ≤ 91.65	(23.3)	97	1.16 (0.86–1.56)	1.28 (0.95–1.74)
≤66.65		99	1.06 (0.78–1.42)	0.98 (0.72–1.34)
Cognitive functioning (≥100.0 as reference)	86.5	72	1.00	1.00
83.33 > CF ≤ 99.99	(18.6)	59	1.18 (0.86–1.62)	1.08 (0.78–1.48)
≤83.32		168	1.29 (0.97–1.73)	1.19 (0.87–1.61)
Social functioning (≥100.0 as reference)	74.7	131	1.00	1.00
66.66 > SF ≤ 99.99	(31.6)	21	1.26 (0.78–2.04)	1.22 (0.75–2.00)
≤66.65		147	1.13 (0.88–1.45)	0.99 (0.77–1.429)
Fatigue (=0 as reference)	43.1	36	1.00	1.00
>0	(28.8)	263	2.08 (1.39–3.11)***	1.73 (1.14–2.61)**
Pain (=0 as reference)	36.3	79	1.00	1.00
>0	(31.2)	220	1.31 (0.99–1.72)	1.10 (0.82–1.47)
Nausea/vomiting (=0 as reference)	11.1	213	1.00	1.00
>0	(21.6)	86	1.16 (0.89–1.51)	1.13 (0.86–1.49)
Dyspnoea (=0 as reference)	34.1	110	1.00	1.00
>0	(32.8)	188	1.33 (1.03–1.71)*	1.22 (0.94–1.58)
Insomnia (=0 as reference)	32.4	120	1.00	1.00
>0	(33.3)	178	1.66 (1.29–2.14)***	1.44 (1.10–1.88)**
Appetite loss (=0 as reference)	36.2	110	1.00	1.00
>0	(34.9)	189	1.40 (1.10–1.80)**	1.42 (1.10–1.84)**
Constipation (=0 as reference)	20.6	179	1.00	1.00
>0	(29.6)	120	1.54 (1.21–1.97)**	1.61 (1.25–2.07)***
Diarrhoea (=0 as reference)	8.9	249	1.00	1.00
>0	(22.4)	50	1.27 (0.92–1.74)	1.14 (0.82–1.57)
Financial difficulties (=0 as reference)	29.3	146	1.00	1.00
>0	(34.6)	153	1.14 (0.89–1.45)	0.99 (0.77–1.27)

* P < 0.05; ** P < 0.01; *** P < 0.001

^a^Adjusted for age, gender, clinical stage (as TNM), and comorbidiy

**Table 3:** Significance of EORTC QLQ-L13 and LCSS scores for overall survival of the lung cancer patients (Univariate and mulitvariate Cox’s regression results)

Variables LC13	Mean (sd)	N	Crude HR (95 % CI)	Adjusted HR^a^ (95 % CI)
Dispnoea (ref = 0)	30.5	235	1.00	1.00
>0	(25.7)	64	0.83 (0.61–1.12)	1.01 (1.00–1.01)
Cough (ref = 0)	39.6	220	1.00	1.00
>0	(32.1)	79	0.93 (0.71–1.22)	1.00 (1.00–1.01)
Hemoptysis (ref = 0)	9.9	67	1.00	1.00
>0	(21.0)	232	1.01 (0.75–1.34)	1.00 (1.00–1.01)
Sore Mouth (ref = 0)	5.8	40	1.00	1.00
>0	(16.3)	259	1.14 (0.79–1.63)	1.00 (0.99–1.01)
Dysphagia (ref = 0)	9.0	54	1.00	1.00
>0	(21.4)	245	0.88 (0.64–1.21)	1.01 (1.00–1.01)
Peripheral neuropathy (ref = 0)	14.0	83	1.00	1.00
>0	(26.3)	216	0.98 (0.75–1.29)	1.01 (1.00–1.01)
Hair loss (ref = 0)	5.9	28	1.00	1.00
>0	(25.7)	269	1.40 (0.91–2.14)	1.00 (0.99–1.00)
Chest pain (ref = 0)	24.5	149	1.00	1.00
>0	(29.7)	150	0.85 (0.67–1.08)	1.00 (1.00–1.01)
Pain in arm or shoulder (ref = 0)	18.1	108	1.00	1.00
>0	(27.7)	191	0.96 (0.74–1.23)	1.00 (1.00–1.01)
Other pain sites (ref = 0)	20.7	186	1.00	1.00
>0	(16.2)	113	1.07 (0.83–1.36)	1.00 (0.99–1.01)
LCSS (Lung Cancer Symptom Scale)				
LCSS index score (30.00 ≤ as reference)	40.5	96	1.00	1.00
30.01 > LCSS.I ≤ 45.99	(20.6)	99	0. 77(0.57–1.05)	0.82 (0.60–1.21)
≥46.00		101	1.16 (0.86–1.55)	1.06 (0.78–1.42)
LCSS symptom score (27.00 ≤ as reference)	38.1	98	1.00	1.00
27.01 > LCSS.S ≤ 43.69	(21.7)	96	0.74 (0.54–1.00)	0.76 (0.56–1.04)
≥43.70		102	1.20 (0.90–1.62)	1.12 (0.83–1.52)

* P < 0.05; ** P < 0.01; *** P < 0.001

^a^Adjusted for age, gender, clinical stage (as TNM), and comorbidiy

**Table 4:** Significance of FACT-L, EQ-5D and ECOG scores for overall survival of the lung cancer patients (Univariate and mulitvariate Cox’s regression results)

Variables FACT-L	Mean (SD)	N	Crude HR (95 % CI)	Adjusted HR^a^ (95 % CI)
Physical (ref = ≥ 23.00)	18.9	90	1.00	1.00
15 > PWB ≤ 22	(6.2)	108	1.37 (1.02–1.84)*	1.21 (0.89–1.63)
≤15		101	1.83 (1.34–2.48)***	1.42 (1.03–1.94)*
Social/Family (ref = ≥ 27.00)	23.5	67	1.00	1.00
20.0 > SWB ≤ 26.99	(4.8)	138	1.19 (0.90–1.57)	1.07 (0.80–1.42)
≤20		94	1.34 (0.73–2.46)	1.37 (0.75–2.54)
Emotional (ref = ≥ 21.00)	17.7	94	1.00	1.00
15 > EWB ≤ 20	(5.3)	94	1.17 (0.87–1.56)	0.97 (0.71–1.33)
≤15		110	1.07 (0.80–1.44)	1.02 (0.75–1.37)
Functional (ref = ≥ 20.00)	17.0	88	1.00	1.00
13 > FWB ≤ 19	(6.3)	90	1.07 (0.80–1.43)	1.12 (0.83–1.51)
≤13		121	1.51 (1.13–2.02)**	1.51 (1.13–2.02)*
Lung Cancer Subscale (ref = ≥ 21.00)	18.7	89	1.00	1.00
15 > LCS ≤ 20	(5.3)	93	0.88 (0.66–1.18)	0.88 (0.66–1.18)
≤15		117	1.52 (1.14–2.03)**	1.42 (1.05–1.92)**
FACT-L (ref = ≥ 105.1)	95.8	97	1.00	1.00
86.99 > FACT-L ≤ 105.1	(20.1)	99	1.20 (0.89–1.61)	1.09 (0.80–1.47)
≤86.99		102	1.63 (1.21–2.19)**	1.31 (0.96–1.79)
FACT-L TOİ^b^ (ref = ≥ 23.00)	54.7	94	1.00	1.00
15 > TOI ≤ 22	(15.2)	103	1.25 (0.93–1.67)	1.18 (0.88–1.59)
≤15		102	1.70 (1.26–2.29)**	1.45 (1.07–1.98)*
FACT-G (ref: ≥ 85.00)	77.1	99	1.00	1.00
46.99 > FACT-G ≤ 62	(16.8)	92	1.32 (0.98–1.74)	1.23 (0.91–1.66)
≤46.99		107	1.61 (1.20–2.16)**	1.29 (0.95–1.76)
Equation5-D				
Mobility (3 as reference)		186	1.00	1.00
1 + 2		113	4.29 (2.67–6.90)***	3.38 (2.05–5.26)***
Self-care (3 as reference)		242	1.00	1.00
1 + 2		57	4.38 (2.14–8.98)***	3.30 (1.56–6.98)**
Usual activities (3 as reference)		167	1.00	1.00
1 + 2		132	1.68 (1.22–2.30)**	1.96 (1.41–2.72)***
Pain (3 as reference)		127	1.00	1.00
1 + 2		172	1.57 (1.07–2.98)*	1.51 (1.03–2.23)*
Mood (3 as reference)		194	1.00	1.00
1 + 2		105	1.11 (0.61–2.04)	(0.54–1.86)
Equation5-D Index (1.0 as reference) 0.65	149	1.00	1.00	
Median value ≥ Index < 1.0 (0.36)	61	1.26 (0.89–1.77)	1.06 (0.75–1.51)	
<Median value	89	1.24 (0.94–1.65)	1.27 (0.95–1.62)	
Equation5-D VAS (≥79.00 as reference) 65.5	101	1.00	1.00	
50.01 > VAS ≤ 78.99 (20.5)	95	1.24 (0.92–1.67)	1.19 (0.83–1.51)	
≤50.00	102	1.40 (1.04–1.87)*	1.17 (0.86–1.60)	
ECOG (Ecog 0 − 1 as reference)	212	1.00	1.00	
ECOG (value 2 + 3 + 4)	87	2.10(1.62–2.73)***	2.01(1.54–2.64)***	

* P < 0.05; ** P < 0.01; *** P < 0.001

^a^Adjusted for age, gender, clinical stage (as TNM), and comorbidiy

^b^A combination of Physical (PWB), Functional (FWB) wellbeing and Lung Cancer Scale (LCS)

Table 4 presents the results of FACT-L, EQ-5D, and ECOG. Physical well-being, functional well-being, LC scales, and the TOI score of the FACT-L had significant impacts on survival, but the physical scale lost its linear trend after adjustment. Moreover, the meaningful impacts of FACT-L and FACT-G scores on survival disappeared after adjustment. All dimensions of EQ-5D, except the mood dimension, had an impact on survival after adjustment for age, gender, clinical stage, and co-morbidities. However, categorised EQ-5D utility and VAS scores showed no effect on survival. The ECOG performance status was the strongest predictor of survival even after adjustment (HR 2.01, 95 % CI 1.54–2.64).

Hierarchical Cox proportional hazard models are presented in Table 5. The results for model A indicated that male gender, younger age (<50 years), stage IV, and co-morbidities were predictors of worse survival. The physical function scale (model B) and the constipation symptom scale (model C) of the QLQ-C30 were significant prognostic factors for survival. In model D, the physical function scale of QLQ-C30 lost its impact on survival, whereas constipation symptom items remained a significant predictor of survival. Model E included EQ-5D dimensions in addition to demographic and clinical variables. The dimensions of mobility and usual activities of the EQ-5D were found to be strong predictors of subsequent survival after adjustment for demographic and clinical variables.

**Table 5:** Contribution of baseline sociodemographic, clinical and quality of life variables for prediction of survival in lung cancer patients

Variables entered	Model A	Model B	Model C	Model D	Model E	Model F
Male (ref: femalef)	1.95 (1.18–3.22)**	2.07 (1.25–3.43)**	2.16 (1.3–3.59)**	2.21 (1.33–3.67)**	1.87 (1.13–3.09)*	1.9 (1.13–3.18)*
Age ref: < 50						
50 ≤ Age < 60	0.76 (0.48–1.22)	0.82 (0.54–1.24)	0.86 (0.54–1.39)	0.89 (0.55–1.44)	0.80 (0.53–1.21)	0.75 (0.46–1.2)
60 ≤ Age < 70	0.64 (0.45–0.92)*	0.91 (0.59–1.4)	0.66 (0.46–0.95)*	0.68 (0.47–0.99)*	0.94 (0.62–1.42)	0.64 (0.44–0.92)*
≥70	0.72 (0.5–1.03)	1.17 (0.72–1.89)	0.74 (0.52–1.07)	0.75 (0.52–1.09)	1.09 (0.68–1.76)	0.66 (0.46–0.96)*
Stage 4 (ref: 3b)	2.05 (1.57–2.68)**	1.83 (1.39–2.42)**	1.85 (1.4–2.45)**	1.75 (1.32–2.33)**	2.13 (1.62-2.82)**	1.94 (1.47–2.55)**
Comorbidity	1.35 (1.04–1.74)*	1.35 (1.04–1.75)*	1.39 (1.07–1.8)*	1.45 (1.11–1.89)**	1.28 (0.98–1.68)	1.38 (1.06–1.8)*
QLQ-C30						
PF(ref: ≥ 86.67)		1.00		1.00		
66.66 > PF ≤ 86.66		1.05 (0.74–1.49)		0.88 (0.6–1.29)		
PF ≤ 66.65		1.52 (1.03–2.24)*		1.24 (0.81–1.88)		
RF(ref: ≥ 100.0)		1.00		1.00		
66.66 > RF ≤ 99.99		1.14 (0.83–1.57)		1.03 (0.75–1.42)		
RF ≤ 66.65		1.26 (0.76–2.07)		1.36 (0.81–2.28)		
FA			1.34 (0.84–2.13)	1.34 (0.8–2.26)		
SL			1.23 (0.92–1.65)	1.22 (0.9–1.65)		
AP			1.1 (0.82–1.48)	1.08 (0.79–1.47)		
CO			1.49 (1.14–1.93)**	1.5 (1.15–1.98)**		
EQ-5D						
Mobility					3.03 (1.65–5.56)**	
Selfcare					0.98 (0.39–2.43)	
Usual activities					1.76 (1.19–2.61)**	
Pain					0.97 (0.62–1.53)	
Mood					0.79 (0.42–1.5)	
FACT-L						
Pwb (ref: ≥ 23.00)						1.00
15 > Pwb ≤ 22						1.24 (0.81–1.91)
Pwb ≤ 15						1.18 (0.83–1.67)
Fwb (ref: ≥ 20.00)						1.00
13 > Fwb ≤ 19						1.24 (0.84–1.84)
Fwb ≤ 13						1.04 (0.74–1.48)
LCS (ref: ≥ 21.00)						1.00
15 > LCS ≤ 20						1.09 (0.76–1.57)
LCS ≤ 15						0.7 (0.51–0.97)*

* P < 0.05; ** P < 0.01

*PF* Physical functioning, *RF* role functioning, *FA* fatigue, *SL* sleep problem, *AP* apetite loss, *CO* constipation, *Pwb* physical wellbeing, *Fwb* functional wellbeing, *LCS* Lung Cancer Scale

**Fig. 1:**
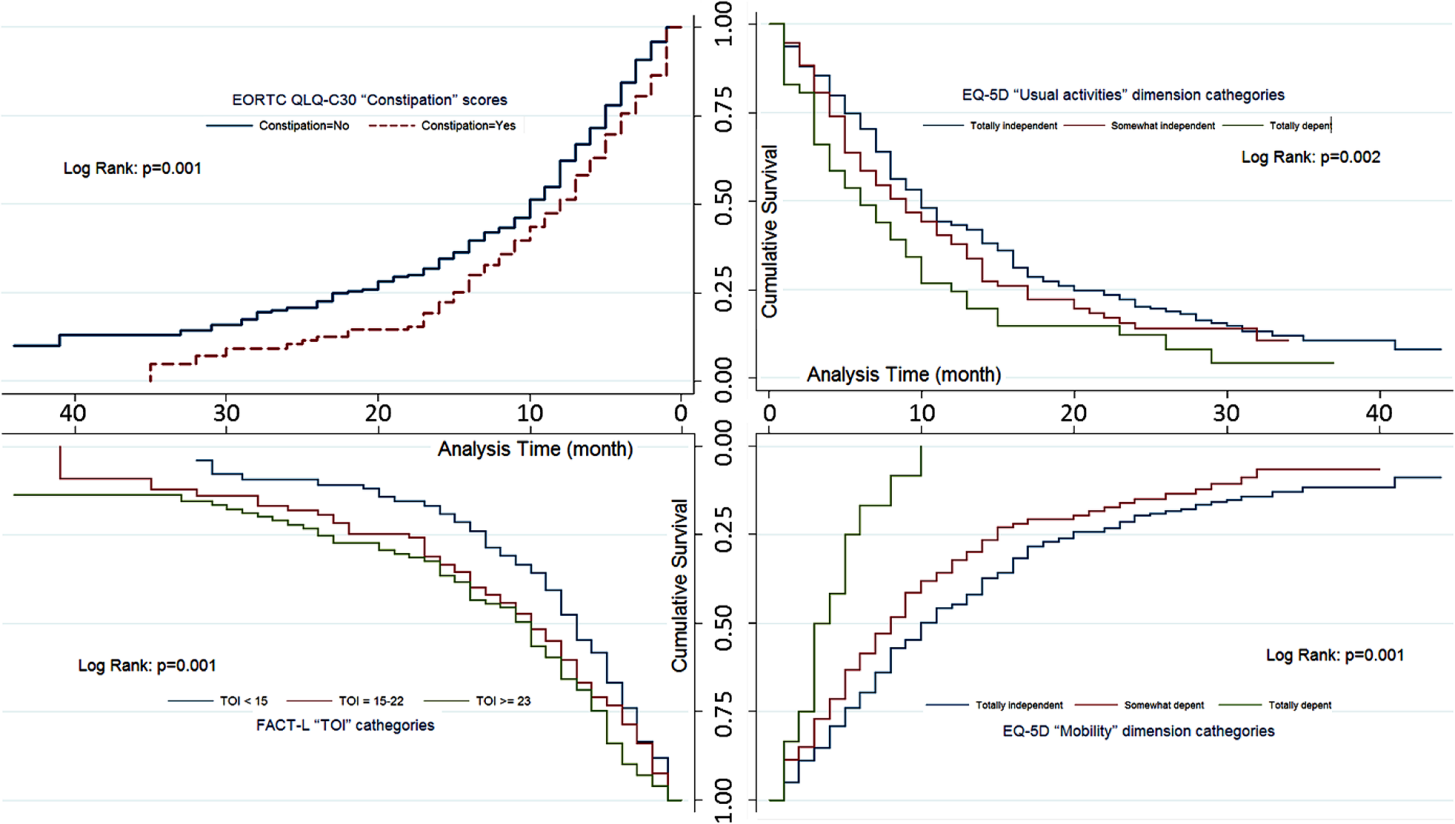
Survival curves

 Survival curves represent meaningful results in the final reduced regression models: In Fig. 1, Kaplan–Meier curves indicate subgroups defined by the QLQ-C30 Constipation scale, and the log-rank test indicated significant differences between the subgroups (P < 0.001). Figure 1 presents the survival curves of the TOI score categories of the FACT-L, which also showed significant differences among the three TOI categories by a log-rank test (P < 0.001). Finally, the dimensions of mobility and usual activities of the EQ-5D subgroups are presented in Fig. 1, indicating meaningful (P < 0.001) subgroup differences between the three dimensional categories.

## Discussion

This study evaluated almost all of the HRQOL tools widely used to assess patient/clinical reported outcomes for their ability to forecast subsequent survival in LC patients.

Before interpreting the effect of HRQOL on survival, we can say that our results confirmed the predictive effects of some known variables on survival, such as gender and cancer stage. Worse survival in males is consistent with the results of Quinten et al. (2014), Efficace et al. (2006), Naughton et al. (2002), and Braun et al. (2011). Although only two stages of cancer (stage 3b and 4) were included in this study, LC stage remained a significant predictor of survival in all models. These results are also consistent with several previous studies (Quinten et al. 2009; Montazeri et al. 2001; Langendijk et al. 2000; Maione et al. 2005; Braun et al. 2011). Our findings also confirmed the dominant predictive value of performance status (measured here by the ECOG) on survival, as reported by many previous studies (Coates et al. 1997; Langendijk et al. 2000; Efficace et al. 2006; Maione et al. 2005; Herndon et al. 1999; Bernhard et al. 1996; Sloan et al. 2012; Qi et al. 2009; Kaasa et al. 1989), even after adjustment for demographic variables and LC stage (HR 2.01, 95 % CI 1.54–2.64).

We found that the presence of any comorbidity was a predictor of worse survival, in contrast to the findings of a phase III Italian study (Maione et al. 2005), which found no significant effect of comorbidities on survival. We assessed comorbidities simply by counting comorbid illnesses, without using any weighted measure, and this may have caused an underestimation of the effect of comorbidities on survival.

Baseline global QOL, physical functioning and role functioning scales and symptom scales for fatigue, insomnia, appetite loss, and constipation of QLQ-C30 were found to be statistically significant prognostic factors for overall survival in patients with LC in this study. QLQ-C30 physical functioning and constipation scores were the strongest predictors of survival in the final reduced model. The findings of this analysis were consistent with those presented in other reports using QLQ-C30 as a predictor of survival (Coates et al. 1997; Quinten et al. 2009, 2014; Grande et al. 2009; Montazeri et al. 2001; Li et al. 2012; Langendijk et al. 2000; Efficace et al. 2006; Maione et al. 2005; Herndon et al. 1999; Polanski et al. 2016; Naughton et al. 2002; Nowak et al. 2004; Nishiyama et al. 2006).

Nowak et al. (2004) and Braun et al. (2011) reported the strongest effect of physical performance on HRQOL among seven studies (Coates et al. 1997; Quinten et al. 2009, 2014; Grande et al. 2009; Herndon et al. 1999; Nowak et al. 2004) that found the physical domain to be a prognostic factor for survival. The physical function was also reported as a strong predictor of survival in a study by Eton et al. (2003), who used FACT-L as a tool of HRQOL.

Our findings confirmed the prognostic ability of the global QOL score of the QLQ-C30 on overall survival (OS), as several studies have demonstrated in advanced LC patients, although some results have been contradictory. In the study by Herndon et al., for example, global QOL was not found prognostic for survival. As Qi et al. (2009) noted, the inclusion of global QOL in the same model with other subscales may lead to multicollinearity, which may be responsible for the inconsistency in the findings. In order to account for this problem we used a different multivariate model. In our model we assessed the global QOL score independent from other scales of the QLQ-C30.

We found the fatigue, appetite loss, insomnia, and constipation symptom scales of the QLQ-C30 to be significant predictors of survival after adjustment in our study. When the generic scales of the QLQ-C30 were included in the models, the constipation scale remained as a unique independent significant predictor of OS. Our findings are consistent with previous reports for appetite loss (Quinten et al. 2009; Grande et al. 2009; Li et al. 2012; Herndon et al. 1999; Polanski et al. 2016) and fatigue (Coates et al. 1997; Montazeri et al. 2001; Li et al. 2012; Herndon et al. 1999; Polanski et al. 2016; Nowak et al. 2004) but not for pain (Quinten et al. 2009, 2014; Li et al. 2012; Efficace et al. 2006; Nowak et al. 2004) or dyspnoea (Coates et al. 1997; Grande et al. 2009; Herndon et al. 1999; Nowak et al. 2004), which have been found to be significant predictors of OS that were not confirmed in our study. No evidence was reported for insomnia in previous work, and just one study (Polanski et al. 2016) showed an effect of constipation, which is consistent with our results.

The physical and functional well-being and LCS of FACT-L were independently significant in the final reduced models, which already accounted for the effects of age, gender, clinical stage (TNM), and comorbidities. Eaton et al. (2003) evaluated FACT-L for its sensitivity on subsequent survival in LC patients in a longitudinal study and found the physical well-being scale to be predictive of survival, consistent with our findings. In a second study by Qi et al. (2009) using the FACT-L, weight loss was found to be the only significant predictor of worse survival and the remaining scales were not predictive. One finding of our study was that LCS had a predictive ability for worse survival when adjusted by demographic and clinical variables, but for longer survival in the final reduced model, which might be due to an inadequate sample size or a colinearity issue when entered into the same model with physical and functional scales. This is why we did not enter TOI in model F, since TOI combines the physical, functional, and LCscales of the FACT-L. Thus, we did not construct an additional multivariate model for TOI score or for ECOG, because they are already presented as the adjusted HRs in Table 4.

As previously confirmed in many studies (Langendijk et al. 2000; Efficace et al. 2006; Maione et al. 2005; Herndon et al. 1999; Bernhard et al. 1996; Reck et al. 2012; Sloan et al. 2012; Qi et al. 2009; Kaasa et al. 1989; Movsas et al. 2009), performance status, assessed by ECOG, was a strong predictor of OS in our study, even after adjustment, indicating the validity of our results.

To our knowledge, our study is the first to forecast subsequent survival using the EQ-5D on OS in cancer patients. All five dimensions of EQ-5D, except the “mood” dimension, were significantly sensitive to subsequent survival in this study even after adjustment. When they were entered into the same multivariate model (model E), the pain and self-care dimensions were excluded from the final reduced model, leaving mobility and usual activities dimensions as strong independent predictors of survival. These two dimensions remained in the model because both refer to “physical independence,” as already confirmed by the findings of QLQ-C30 and FACT-L in this study. In fact, Jang et al. (2010) demonstrated that QLQ-C30 data could be used to derive EQ-5D utility scores in their study, but EQ-5D findings differed from those of others by their very high HRs, indicating EQ-5D dimensions as important predictors of OS.

In this study, we included a battery of HRQOL instruments to evaluate predictors of survival. Among them, none of the LC13 scales or LCSS index or symptom scores were found to be predictive of survival. These results are consistent with findings in previous studies (LC13: Montazeri et al. 2001; Li et al. 2012; Polanski et al. 2016; Nowak et al. 2004, LCSS: Qi et al. 2009).

This study has some limitations. One is that this study merged all types of lung cancer, and the findings could not be expressed purely for any specific type of lung cancer. Second, we could not use the “duration of cancer” variable in this study, because most of the patients were newly diagnosed, but this may be strength of the study too. A third limitation was the lack of FLIC scale in the questionnaire battery, due to the unavailability of a validated Turkish version (Ganz et al. 1991). Finally, we could not run stratified analyses of the treatment arms due to the sample size, so the conclusions of this study may not be generalisable to all types of LC or treatments.

## Conclusions

HRQOL serves as an additional predictive factor for survival that supplements ‘traditional’ clinical factors, such as age, gender, stage, and comorbidity in LC patients. Besides the strong predictive ability of the ECOG performance status on survival, FACT-L and the Eq5D were the most promising QOL instruments for the purpose in this study.
